# Healthcare workers' and parents' perceptions of measures for improving adherence to hand-hygiene

**DOI:** 10.1186/1471-2458-11-466

**Published:** 2011-06-13

**Authors:** Marta L Ciofi degli Atti, Alberto E Tozzi, Gaetano Ciliento, Manuel Pomponi, Silvia Rinaldi, Massimiliano Raponi

**Affiliations:** 1 Medical Direction, Bambino Gesù Children's Hospital, Piazza S. Onofrio 4, 00165 Rome, Italy; 2 Epidemiology Unit, Bambino Gesù Children's Hospital, Piazza S. Onofrio 4, 00165 Rome, Italy

## Abstract

**Background:**

This study was conducted to evaluate perceptions of healthcare workers (HCW) and parents regarding hand-hygiene and effectiveness of measures for increasing hand-hygiene adherence, in a children's hospital in Italy.

**Methods:**

A cross-sectional study was performed from 5 to 13 July 2010, using two self-administered anonymous questionnaires (one for HCWs and one for parents/caregivers). The questionnaires included information regarding individual perceptions associated with hand hygiene.

**Results:**

We collected 139 questionnaires from HCWs and 236 questionnaires from parents. Alcohol-based handrub was reported to be available at the point of care by 95.0% of the HCWs and in the child's room by 97.0% of the parents. For both HCWs and parents, availability of alcohol-based handrub was perceived as the most useful action for improving adherence to hand hygiene (scores ≥ 6 on a 7-point Likert-type scale: 84.8% [CI95%78.0-90.1] for HCWs and 87.9% [CI95% 83.3-91.7] for parents). Parents' reminding HCWs to perform hand hygiene was perceived as the least useful action (scores ≥ 6: 48.9% [CI95% 40.5-57.3] for HCWs and 55.7% [CI95% 49.2-62.1] for parents). Factors that affected HCWs' perceptions of the effectiveness of actions for improving adherence to hand hygiene included years of practice, type of ward and previous formal training on hand hygiene. For parents, factors affecting perceptions included previous information on hand hygiene and previous hospitalizations for their child.

**Conclusions:**

Investigating HCWs' and parents' perceptions of measures for improving adherence can provide useful information for implementing actions for hand-hygiene promotion in children's hospitals. In this study, HCWs' and parents' perceptions were similar; alcohol-based hand-rub availability was perceived as the most useful tool, confirming its crucial role in multimodal interventions. Poor perception of inviting parents to remind HCWs to perform hand-hygiene has been previously observed, and deserves further investigation. Information and education activities were associated with more positive perceptions regarding various improvement measures. Though the relationship between perceptions and behaviours remains to be fully determined, HCWs should participate in formal training and families should be properly informed, not only to increase knowledge but also to improve perceptions on effectiveness of actions to be implemented.

## Background

Hospital acquired infections (HAI) represent one of the greatest risks associated with health care [1]. An estimated 5-15% of hospital patients acquire an infection during hospital stay [2], and the estimated annual economic burden is €13-24 billion in Europe and $6.5 billion in the United States [3]. However, it has been reported that at least 20% of all HAIs are preventable through infection-control measures applied under routine working conditions [4]. Of these measures, hand hygiene (i.e., handwashing with either plain or antiseptic soap and water or alcohol-based products) is frequently cited as the single most important means of preventing the transmission of infectious agents [5].

Nonetheless, only 50-70% of health care workers (HCW) comply with hand-hygiene recommendations [6-9]. Adherence to recommendations is influenced by knowledge, perceived risk, individual attitude, accessibility of hand-hygiene agents, workload and type of ward [3,5-10]. Whereas most of the literature focuses on the hand-hygiene practices of HCWs, few studies have assessed patients' knowledge and attitudes and their perception of their own role in improving HCW adherence [11-13]. In paediatric healthcare settings, parents play a major role in promoting patient safety, given that children may not be capable of bringing risk to the attention of healthcare providers. Families are thus an important component of multimodal approaches to hand-hygiene improvement [14].

In the present study, we evaluated perceptions of both HCWs and the parents of hospitalized children regarding hand-hygiene and perceived effectiveness of measures for increasing hand-hygiene adherence in a children's hospital in Italy.

## Methods

### Setting

The Bambino Gesù Children's Hospital (*Ospedale Pediatrico Bambino Gesù*; OPBG) is a tertiary care research hospital in Rome, Italy. It is the largest children's hospital in Italy and has a total inpatient bed capacity of 607. In 2009, there were 30,344 inpatient admissions, with a mean length of stay of 5.9 days. During hospitalization, one parent/caregiver is invited to stay with the child all day long, while other relatives and visitors can stay in patients' rooms during wards' visiting hours.

The OPBG was first accredited by Joint Commission International in 2006, and re-accredited in 2009. In 2008, actions recommended by the WHO multimodal strategy for promoting handwashing [3] were implemented. Posters and leaflets for parents were distributed in the wards; alcohol-based handrub was made available at the point of care; and staff training was conducted. Furthermore, the adherence of HCWs to hand hygiene was directly monitored by infection-control nurses, and data feed-back was provided quarterly.

### Study design

We performed a cross-sectional study from 5 to 13 July 2010 using two self-administered anonymous questionnaires (one for HCWs and one for parents/caregivers). The questionnaires were distributed in each ward by the hospital infection-control nurse to HCWs and to the parent/caregiver who was with the child all day long, and returned on the same day. Participation was on a voluntary basis. The study was part of the hospital's activities for HAI prevention and control and was reviewed and approved by the Hospital Infection Prevention and Control Committee.

### Questionnaires

The HCW questionnaire was based on the Perception Survey for Health-Care Workers of the WHO Cleanyourhands campaign [15]. It included: demographic data; information on profession; previous formal training in hand hygiene; perceptions regarding a number of factors, in particular, the impact of HAI on the patient's clinical outcome, the effectiveness of hand hygiene in preventing cross transmission, the importance of hand hygiene as a safety objective in the ward, the availability of alcohol-based handrub at the point of care, the easiness of use of alcohol-based handrub, the tolerance of alcohol-based handrub; own adherence to hand hygiene (expressed as the percentage of situations in which the respondent perceived that he/she actually performed hand hygiene; from 0% to 100%), the adherence of other HCWs to hand-hygiene practices (expressed as the percentage of situations in which, according to the respondent's perception, other HCWs actually performed hand hygiene; from 0% and 100%), and the perceived effectiveness of various measures for improving hand hygiene. Question regarding perceived effectiveness of improvement measures was: "In your opinion, how effective are the following interventions to increase compliance with hand hygiene?". The response format for questions on the perceptions of the effectiveness of practices for improving hand-hygiene consisted of a 7-point Likert-type scale.

The parent questionnaire was based on the HCW questionnaire and included: demographic data; information on whether or not the parent had received information on hand hygiene; perceptions regarding the risk of acquiring an HAI, the importance of hand hygiene for healthcare safety, the effectiveness of hand hygiene in preventing cross transmission; the availability of alcohol-based handrub in the patient's room; the easiness of use of alcohol-based handrub, their tolerance of alcohol-based handrub, and the perceived effectiveness of various practices for improving hand hygiene (7-point Likert-type scale).

### Data analysis

The responses to the questionnaires were entered in an Epi-info database. The statistical analysis was performed using Stata version 10.0. For the univariate analyses, χ2 or Fisher exact tests were used for discrete variables, and the Wilcoxon rank-sum test was used for continuous variables; 95% Confidence Intervals (95%CI) were calculated with MidP-exact test. For HCWs, gender, specific profession, number of years of practice, type of unit (medical/surgical wards, intensive care units) and previous formal training in hand-hygiene were analyzed as potential determinants of perceptions using multiple logistic regression modelling. For parents, the potential determinants of perceptions consisted of gender, whether the individual was the child's mother, father or a non-parental caregiver, type of unit, previous child's hospitalization, and whether or not the parent had received information on hand hygiene. In the multiple logistic regression, the perceptions of the effectiveness of actions for improving hand-hygiene were considered as outcomes; in the analysis, responses in the 7-point Likert-type scale were categorized as dichotomous variables (< 6; ≥ 6).

## Results

We collected 139 questionnaires from HCWs and 236 questionnaires from parents or caregivers. The mean and median age of HCWs was 37 years (range: 23-64 years); the mean and median number of years of practice were 11.1 and 8.0, respectively (range: 1-37 years). The characteristics of the study participants are reported in Table 1.

**Table 1:** Characteristics of healthcare workers and parents participating in the study

Healthcare Workers (No. 139)			Parents/Caregivers (No. 236)		
	**No**	**%**		**No**	**%**

Females	101	72.7	Females	170	72.5

Professional category			Caregiver category		

Physician	41	29.5	Mother	170	72.5

Nurse	91	65.5	Father	50	21.5

Other	7	5.0	Other	18	6.0

Type of ward			Type of ward		

Surgical/medical	106	76.3	Surgical/medical	206	87.3

Intensive care unit (ICU)	33	23.7	ICU	30	12.7

Hand-hygiene formal training			Hand-hygiene information		

Yes, at Bambino Gesù Hospital	64	46.0	Yes, at Bambino Gesù Hospital	125	53.0

Yes, outside Bambino Gesù Hospital	10	7.2	Yes, outside Bambino Gesù Hospital	76	32.2

No	65	46.8	No	35	14.8

Years of practice^			First hospitalization for child		

≤ 5	58	43.9	Yes	99	41.9

>5	74	56.1	No*	137	58.1

^ Total number of respondents = 132

* mean number of previous hospitalizations = 5.0; median number of previous hospitalizations = 3.0 (range: 1-30)

Alcohol-based handrub was reported to be available at the point of care by 95.0% (132/139) of the HCWs and in the child's room by 97.0% (229/236) of the parents. It was reported to be very easy to use (score 6-7) by 91.2% (125/137) of the HCWs and 90.6% (212/234) of the parents; 51.5% (70/136) of the HCWs and 80.3% (188/234) of the parents reported that the alcohol-based handrub was very tolerable (score 6-7). According to the multiple logistic regression, none of the characteristics of the HCWs or parents were significantly associated with ease of use or tolerability. The majority of HCWs and parents had received formal training or information on hand hygiene, in most cases at the hospital itself (Table 1). Hand hygiene was considered to be highly/very highly effective in preventing HAIs by the vast majority of respondents (Table 2).

**Table 2:** Healthcare workers' (HCW) and parents' perceptions of the impact of hospital-acquired infections (HAI) and of the importance of hand hygiene

Healthcare Workers			Parents		
	**No**	**% (95%CI)**		**No**	**% (95%CI)**

Impact of HAIs on patient outcome (high/very high)	114/132	86.4 (79.7-91.4)	Risk of acquiring an HAI (high/very high)	159/234	67.9 (61.8-73.7)

Hand hygiene effectiveness in preventing HAIs (high/very high)	135/138	97.8 (94.2-99.4)	Hand-hygiene effectiveness in preventing HAIs (high/very high)	209/232	90.1 (85.7-93.5)

Importance in the ward of hand hygiene with respect to all patient safety issues (high/very high)	136/138	98.5 (95.3-99.8)	Importance of hand hygiene for healthcare safety (high/very high)	230/235	97.9 (95.3-99.2)

Importance that the head of the ward places on the fact that HCWs perform optimal hand hygiene (high/very high)	94/135	69.6 (61.5-76.9)	Importance that the head of the ward places on the fact that parents perform optimal hand hygiene (high/very high)	138/226	61.1 (54.6-67.3)

Effort required to perform good hand hygiene when caring for patients (high/very high)	82/137	59.8 (51.5-67.8)	Effort required to perform good hand hygiene (high/very high)	154/233	66.1 (59.8-72.0)

The adherence to hand hygiene in the hospital, as perceived by HCWs, was 76.8% (range: 20-100%), whereas the HCWs' perceived self-adherence was 84.6% (range: 20-100%) (p < 0.0001).

The perceptions of HCWs and parents regarding the effectiveness of measures for improving adherence to hand hygiene are reported in Table 3. For both HCWs and parents, the highest score was for availability of alcohol-based handrub, whereas the lowest score was for parents' reminding HCWs to perform hand hygiene.

**Table 3:** Healthcare workers' and parents' perceptions of the effectiveness of measures for improving hand hygiene; number and proportion of answers with scores ≥ 6

Health Care Workers			Caregivers		
	**No**	**% (95%CI)**		**No**	**% (95%CI)**

Leaders and senior managers support and openly promote hand hygiene	85/137	62.0 (53.7-69.9)	Physicians support and openly promote hand hygiene	164/231	71.0 (64.9-76.6)

Alcohol-based handrub always available at each point of care	117/138	84.8 (78.0-90.1)	Alcohol-based handrub always available at each point of care	205/233	87.9 (83.3-91.7)

Hand-hygiene posters are displayed at point of care as reminders	101/138	73.7 (65.9-80.6)	Hand-hygiene posters are displayed at point of care as reminders	190/234	81.2 (75.8-85.8)

Each healthcare worker receives education on hand hygiene	107/138	77.5 (70.0-83.9)	Each parent/caregiver receives information on hand hygiene	155/232	66.8 (60.6-72.6)

Clear and simple instructions for hand hygiene are made visible for every healthcare worker	105/138	76.1 (68.4-82.6)	Clear and simple instructions for hand hygiene are made visible for every parent/caregiver	161/233	69.1 (62.9-74.8)

Patients/parents are invited to remind healthcare workers to perform hand hygiene	66/135	48.9 (40.5-57.3)	Patients/parents are invited to remind healthcare workers to perform hand hygiene	126/226	55.7 (49.2-62.1)

Healthcare workers regularly receive feedback on their hand hygiene performance	86/138	62.3 (54.0-70.1)			

You always perform hand hygiene as recommended (being a good example for your colleagues)	96/135	71.1 (63.0-78.3)			

According to the multiple regression, the most important determinant of HCWs' perceptions of the effectiveness of measures for improving adherence to hand hygiene was the number of years of practice (Table 4): being employed for more than 5 years was significantly associated with a better perception of the effectiveness of the support of leaders and senior managers, of the availability of alcohol-based handrub, of the visibility of clear and simple instructions, and of the feed-back to HCWs on their hand hygiene performance. Having received formal training was associated with better perception of the effectiveness of education on hand hygiene, hand-hygiene posters, and the feed-back to HCWs on their hand hygiene performance. HCWs in medical/surgical wards had a better perception than HCWs in ICUs regarding the effectiveness of education on hand-hygiene and visibility of clear and simple instructions.

**Table 4:** Association between characteristics of healthcare workers (HCW) and perceptions of the effectiveness of measures for improving adherence to hand hygiene^

	Support of leaders and senior managers			Alcohol-based handrub availability			Hand-hygiene posters displayed			Hand-hygiene education/information		
	**No. and % answers with score ≥ 6**	**p-value**	**Adjusted p-value**	**No. and % answers with score ≥ 6**	**p-value**	**Adjusted p-value**	**No. and % answers with score ≥ 6**	**p-value**	**Adjusted p-value**	**No. and % answers with score ≥ 6**	**p-value**	**Adjusted p-value**

Gender												
Male	17 (58.6)	NS	NS	27 (90.0)	NS	NS	19 (63.3)	NS	NS	22 (73.3)	NS	NS
Female	63 (63.0)			82 (82.0)			75 (75.8)			79 (79.0)		

Professional category												
Physician	24 (58.5)	NS	NS	36 (87.8)	NS	NS	26 (63.4)	NS	NS	28 (68.3)	NS	NS
Nurse	55 (61.8)			74 (82.2)			70 (78.7)			73 (81.1)		

Years of practice												
≤5	25 (43.9)	0.000	0.001	44 (75.9)	0.022	0.023	37 (63.8)	0.016	NS	43 (74.1)	NS	NS
>5	54 (74.0)			66 (90.4)			59 (81.9)			60 (82.2)		

Type of Unit												
Medical/surgical	68 (65.4)	NS	NS	89 (84.8)	NS	NS	79 (76.0)	NS	NS	87 (82.9)	0.009	0.002
ICU	17 (51.5)			28 (84.4)			22 (66.7)			20 (60.6)		

Previous formal training												
Yes	50 (68.5)	NS	NS	61 (82.4)	NS	NS	61 (83.6)	0.005	0.006	67 (90.5)	0.000	0.006
No	35 (54.7)			56 (87.5)			40 (62.5)			40 (62.5)		

	**Visibility of clear and simple instructions**			**Patients/parents invited to remind HCWs to perform hand hygiene**			**Feedback to HCWs on their hand hygiene performance**			**Being a good example for colleagues**		

	**No. and % answers with score ≥ 6**	**p-value**	**Adjusted p-value**	**No. and % answers with score ≥ 6**	**p-value**	**Adjusted p-value**	**No. and % answers with score ≥ 6**	**p-value**	**Adjusted p-value**	**No. and % answers with score ≥ 6**	**p-value**	**Adjusted p-value**
Gender												
Male	21 (70.0)	NS	NS	23 (76.7)	0.049	NS	18 (64.3)	NS	NS	18 (60.0)	NS	NS
Female	76 (76.0)			58 (58.0)			72 (72.7)			43 (44.3)		

Professional category												
Physician	28 (68.3)	NS	NS	27 (65.9)	NS	NS	26 (66.7)	NS	NS	19 (46.3)	NS	NS
Nurse	72 (80.0)			54 (60.0)			65 (73.0)			44 (50.0)		

Years of practice												
≤5	38 (65.5)	0.015	0.016	31 (53.4)	NS	NS	33 (57.9)	0.003	0.004	27 (48.2)	NS	NS
>5	61 (83.6)			50 (68.5)			58 (81.7)			37 (50.7)		

Type of Unit												
Medical/surgical	86 (81.9)	0.006	0.001	63 (60.0)	NS	NS	77 (74.8)	NS	NS	53 (52.0)	NS	NS
ICU	19 (57.6)			23 (69.7)			19 (54.9)			13 (39.4)		

Previous formal training												
Yes	59 (79.7)	NS	NS	48 (64.9)	NS	NS	57 (79.2)	0.022	0.041	33 (46.5)	NS	NS
No	46 (71.9)			38 (59.4)			39 (61.9)			33 (51.6)		

^ denominators used for calculating percentages vary from cell to cell, according to the number of respondents

According to the multiple regression, among parents, the determinants of perceptions of the effectiveness of actions for improving adherence to hand hygiene were: previous hospitalizations for their children, which was associated with a poorer perception of the usefulness of the parents' reminding HCWs to perform hand hygiene (score 6-7: 45.9% vs. 63.3% for parents whose child was hospitalized for the first time; p = 0.008); and previous information on hand hygiene, which was associated with a better perception of both the usefulness of providing information to parents (score 6-7: 70.6% vs. 45.7% for parents who did not receive information; p = 0.005) and of the visibility of clear and simple instructions (score ≥6: 72.7% vs. 48.6% for parents who did not receive information, p = 0.005).

## Discussion

Investigating perceptions of the effectiveness of measures for improving hand hygiene is a key factor in promoting adherence, since it can help to implement interventions that are perceived as most effective. Parents' involvement in children's care is essential in promoting patient safety, including proper hand hygiene [14]. To the best of our knowledge, this is the first study in which the perceptions of HCWs and parents of hospitalized children on hand hygiene were simultaneously assessed. Interestingly, the results were similar for the two groups, both of which perceived that the availability of alcohol-based handrub was the most useful measure and that inviting parents to remind HCWs to perform hand hygiene was the least useful one.

Alcohol-based handrub reduces bacterial microflora of hands [16], increases hand-washing adherence and frequency [17,18], and decreases the occurrence of nosocomial infections [19]. The availability of a hand-rub solution at the point of care is also a strong predictor of physicians' adherence to hand hygiene [6]. Thus the availability of handrub has greatly modified hand-hygiene practices and is now considered to be a standard of care [3]. In our study, the high perception of its usefulness in improving adherence confirms that it is a crucial part of multimodal interventions for promoting hand hygiene. The high proportion of HCWs and parents reporting that alcohol-based handrub was available is also reassuring.

However, in interpreting our results it should be considered that the study was conducted in early summer 2010, after the 2009-2010 A(H1N1) influenza pandemic and the public-health campaigns for promoting interventions for reducing viral transmission, which included recommendations for hand-washing [20]. These campaigns, together with the information provided by the mass media and public-health agencies, could have contributed to improving the perception of the usefulness of alcohol hand-rub.

Nonetheless, our results stress that the parents' role in improving the hand hygiene of HCWs deserves attention. Various studies have shown that patients are not very willing to ask a nurse or physician to perform hand hygiene or to verify if they have washed their hands [13,21,22]. Furthermore, of the diverse scenarios for reducing medical errors, the one in which the patient asked HCWs if they had washed their hands was perceived to be the least useful and the least likely to be undertaken by patients [23]. The willingness of patients to question health professionals' actions is probably influenced by a number of factors, including the geographical setting, socio-demographic factors, and personality traits, in addition to the way in which parents are asked about this willingness [13,24,25].

According to our multiple logistic regression, none of the HCWs' characteristics were affected the perception of the usefulness of parents' reminding HCWs to wash their hands, whereas the only factor that affected parents' perception of the effectiveness of this measure was a previous hospitalization for their child. In particular, parents of children who had been previously hospitalized had a poorer perception than parents whose child was hospitalized for the first time. However, given that we did not collect information on the diagnosis at admission, we were not able to evaluate the role of underlying diseases in hospitalized children. In fact, parents of children with chronic diseases that require multiple hospitalizations could have different perceptions regarding their interactions with HCWs [26], compared with parents of children hospitalized for acute conditions. Nonetheless, the finding that parents of previously hospitalized children had a poorer perception of the usefulness of parents' reminding HCWs to wash their hands is worrisome, since empowering parents is even more important for children with frequent access to health services, since these children have a greater risk of acquiring nosocomial infections.

Informing patients upon hospital admission that they should ask HCWs to wash their hands can be effective in increasing the adherence of HCWs to proper hand hygiene [12,27]. In studies in which patients were advised to do so, upon discharge 90-100% of them confirmed having asked a nurse [12,27] and 31-35% a physician [11,12]. However, none of these studies explored the attitudes of parents of hospitalized children. For the parents in our study, having received information on hand hygiene did not influence their perception of the usefulness of parents' reminding HCWs to wash their hands. However, of interest is the finding that parents who had received information had a better perception of the effectiveness of parent information in improving adherence. Delivering information on hand hygiene to all parents of hospitalized children is thus crucial to increasing their awareness about hand hygiene and to improve perceptions on actions taken for improvement.

In our study, more positive perceptions were reported by HCWs with more than 5 years of practice (compared to ≤5 years), those with formal training (compared to those without), and those in medical/surgical wards (compared to those in ICUs). In particular, HCWs with more than 5 years of practice showed a more positive perception of measures involving management, the environmental context, education and information (i.e., leaders and senior managers' support, the availability of alcohol-based handrub, hand-hygiene education, the visibility of clear and simple instructions, and the feedback of data on hand-hygiene adherence). HCWs with previous formal training and working in medical/surgical wards had a more positive perception of education and information activities (i.e., display of posters on hand hygiene, visibility of clear and simple instructions, hand-hygiene education, and the feedback of data on hand-hygiene adherence). The complex relationship between HCWs and hand hygiene requires that intervention strategies for promoting this behaviour be multimodal [3]. Our results suggest that attending formal training on hygiene induces a positive perception in HCWs regarding a series of measures.

Our study has several limitations. Since it was carried out on a voluntary basis, there may have been a selection bias. Furthermore, when information on behaviour is self-reported, respondents tend to overscore socially desirable behaviour, which can lead to adherence's being overestimated by up to three times [28,29]. Respondents can also have unrealistic estimations of their own behaviour [5,11,12], as shown by the discrepancy between the HCWs' perceived adherence to hand hygiene in the hospital and the reported personal adherence to hand hygiene (76.8% vs. 84.6%; p < 0.0001). Moreover, HCWs can believe that they wash their hands when necessary even when observations indicate otherwise [30,31].

## Conclusions

Investigating HCWs' and parents' perceptions of measures for improving adherence can provide useful information for designing and implementing tailored actions for children's hospitals.

In this study, HCWs' and parents' perceptions were similar; alcohol-based hand-rub availability was perceived as the most useful tool, confirming its crucial role in multimodal interventions. Poor perception of inviting parents to remind HCWs to perform hand-hygiene has been previously observed, and deserves further investigation. Information and education activities were associated with more positive perceptions regarding various improvement measures. Though the relationship between perceptions and behaviours remains to be fully determined [32,33], all HCWs should participate in formal training and all families should be properly informed on hand hygiene, not only to increase knowledge and possibly to increase adherence [7,11], but also to improve perceptions on effectiveness of actions to be implemented.

## Competing interests

The authors declare that they have no competing interests.

## Authors' contributions

MCDA contributed to the conception of study and interpretation, and writing the manuscript. AET participated in the statistical analysis, interpretation, and writing the manuscript. GC participated in the design and data collection. MP and SR participated in the data collection. MR participated in the design, and interpretation. All authors read and approved.

## Pre-publication history

The pre-publication history for this paper can be accessed here:

http://www.biomedcentral.com/1471-2458/11/466/prepub
